# Association between Water and Energy Requirements with Physical Activity and Fat-Free Mass in Preschool Children in Japan

**DOI:** 10.3390/nu13114169

**Published:** 2021-11-21

**Authors:** Yosuke Yamada, Hiroyuki Sagayama, Jun Yasukata, Akiko Uchizawa, Aya Itoi, Tsukasa Yoshida, Daiki Watanabe, Yukako Hashii-Arishima, Hisashi Mitsuishi, Makoto Nishimura, Misaka Kimura, Yoshiko Aoki

**Affiliations:** 1 Institute for Active Health, Kyoto University of Advanced Science, Kyoto 621-8555, Japan; yamaday@nibiohn.go.jp (Y.Y.); t-yoshida@nibiohn.go.jp (T.Y.); d2watanabe@nibiohn.go.jp (D.W.); kimura.misaka@kuas.ac.jp (M.K.); 2 National Institute of Health and Nutrition, National Institutes of Biomedical Innovation, Health and Nutrition, Tokyo 162-8636, Japan; aitoi@kwjc.kobe-wu.ac.jp (A.I.); yukako.arishima@gmail.com (Y.H.-A.); 3 Faculty of Health and Sport Sciences, University of Tsukuba, Ibaraki 305-8574, Japan; sagayama.hiroyuki.ka@u.tsukuba.ac.jp; 4 Department of Sports and Health Sciences, Faculty of Human Sciences, University of East Asia, Yamaguchi 751-8503, Japan; yasukata@toua-u.ac.jp; 5 Graduate School of Comprehensive Human Science, University of Tsukuba, Tsukuba 305-8574, Japan; s2030446@s.tsukuba.ac.jp; 6 Faculty of Health and Welfare, Kobe Women’s University, Hyogo 650-0046, Japan; 7 Faculty of Health and Medical Sciences, Kyoto University of Advanced Science, Kyoto 621-8555, Japan; mitsuishi.hisashi@kuas.ac.jp; 8 Faculty of Education, Bukkyo University, Kyoto 603-8301, Japan; m-nishimura@bukkyo-u.ac.jp

**Keywords:** children, doubly labeled water, exercise, Japan, prediction equation, preschool, total energy expenditure, water turnover

## Abstract

Water and energy are essential for the human body. The doubly labeled water (DLW) method measures water turnover (WT) and total energy expenditure (TEE), which serves as a benchmark for the adequate intake (AI) of water and estimated energy requirements (EER). The objective of the current study was to examine the association of WT and TEE with physical activity and body composition in Japanese preschool children. We included 41 preschool children (22 girls, 19 boys) aged 3–6 in this study. WT, TEE, and fat-free mass (FFM) were obtained using DLW. Physical activity was measured using a triaxial accelerometer and categorized as light (LPA; 1.5–2.9 Metabolic equivalents, METs) and of moderate-to-vigorous intensity (MVPA; ≥3.0 METs). Exercise duration (Ex) was defined as ≥4.0 METs of physical activity. WT and TEE moderately positively correlated with Ex, but not with LPA. WT moderately positively correlated with BW and FFM while TEE strongly. We established predictive equations for WT and TEE using body weight (BW), FFM, step count, and Ex to guide the AI of water and EER in Japanese preschool children. We found that FFM and step count are the determinants of TEE, and that BW and Ex are the determinants of WT in preschool children.

## 1. Introduction

Water and energy are required by the human body as essential nutrients [1,2,3]. Humans can only survive for a few days in the absence of water. The human body also uses energy from food sources for proper body functioning. Growth and development of children is hindered by malnutrition; poor energy intake brings about stunting and wasting, while excessive energy intake is a leading factor in overweight and obesity [4]. Therefore, determining the adequate intake (AI) of water and estimated energy requirement (EER) is vital for children.

The doubly labeled water (DLW) method is a technique that measures water turnover (WT) [5] and total energy expenditure (TEE) [6], which are the benchmark for the AI of water and EER, respectively. The DLW technique is costly and time wasting, which makes the method limited for research purposes. Therefore, more convenient and practical variables are needed in the clinical setting to establish prediction equations for WT and TEE.

To the best of our knowledge, no study has reported WT in Japanese preschool children using the DLW method. The present study aims to investigate the association between WT and TEE with physical activity and fat-free mass (FFM) in preschool children, 4–6 years of age, in Japan. Categorization of physical activity is by its intensity: light (LPA; 1.5–2.9 metabolic equivalents (METs)) and medium-to-high intensity (MVPA; ≥3.0 METs). Accelerometers are used to track the intensity and time of physical activity, and past studies defined the duration of exercise as ≥4.0 METs of physical activity [7]. We made the hypothesis that, in preschool children, WT and TEE are associated with exercise duration rather than LPA.

Many previous studies examined the estimative models of TEE in children [8,9], but few studies examined the estimative models of water turnover in children. Water turnover measured by the stable isotope method serves as a benchmark for the AI of water. However, the stable isotope method is costly and time wasting, which makes the method limited for research purposes. Therefore, obtaining an equation to estimate water turnover is important to predict the AI of water in preschool children.

## 2. Materials and Methods

### 2.1. Participants

We used 41 healthy young children, between the ages of 3 and 6 as participants. The criteria for inclusion were the absence of a short or long-term illness or injury and well-informed parental agreement to take part in this experiment. An electric scale measured the body weight rounded to the closest 0.1 kg with the children wearing light clothes. Upright height was recorded to the closest 0.1 cm using a stadiometer with the participants barefoot.

### 2.2. Doubly Labeled Water

WT and TEE measurement was undertaken with the DLW technique for a week. Upon accessing the preschool at 0 days, a urine specimen was acquired to measure the baseline ^2^H and ^18^O prior to administering DLW. A prior mixed dose of nearly 0.12 g/kg approximated total body water (TBW) of ^2^H_2_O (99.8 at.%; Taiyo Nippon Sanso, Tokyo, Japan) and 2.5 g/kg approximated TBW of H_2_^18^O (10 at.%; Taiyo Nippon Sanso) was consumed by each participant [10]. Collection of urine samples was carried out the following day (day 1) and on day 8. The urine specimens were then kept in storage at a freezing temperature of −15 °C to be analyzed at a future date using isotope ratio mass spectrometry (Hydra 20–20; Sercon, UK). Previously, detailed investigation of isotope mass spectrometry use was explained [10,11].

Determination of the dilution space of ^2^H and ^18^O (Nd and No, respectively) was carried out by dividing the amount of the given tracer using the intercept method in two specimens from the first day and two samples from the eighth day. From the baseline, all day 8 specimens exceeded 8‰. The present study’s Nd/No of the (1.037 ± 0.012) was similar to that of past studies [12,13]. Thus, TBW calculation in (g) was carried out through the mean of the obtained result when Nd was divided by 1.043 and No was divided by 1.007. TBW (mol) and is found at TBW (g)/18.02. The disposed-of rates of ^2^H and ^18^O (kd and ko, respectively) were concluded, and the calculation of the production rate of carbon dioxide (rCO_2_) (mol/d) was at 0.4554 × TBW (mol) × (1.007 × ko − 1.043 × kd), assuming that fractionation of the isotope only draws water to breath using Equation (A6) by Schoeller et al. [14] and the dilution space revised constant is as it is used by Sagayama et al. [13]. Determination of the rCO_2_ (L/d) was at 22.26 × rCO_2_ (mol/d). Through assumption, we determined the respiratory quotient (RQ) at 0.87 [15], and the Weir’s equation was modified to calculate TEE [16] this way: TEE (kcal/d)  =  1.106 × rCO_2_ + 3.94 × (rCO_2_/RQ). The International Atomic Energy Agency (IAEA) describes quality checklists [17]. The TBW with the hydration factor for children is age-dependent and calculated FFM [18]. FFM and body weight were used to calculate fat mass (FM) and body fat percentage (%fat). Calculation of WT was as follows: WT = kd × Nd.

The Recommended Dietary Allowances for Japanese equation was used to calculate the predicted basal metabolic rate (BMR) [19] as in past studies [11], which examined the BMR calculations in the Japanese population systematically to come up with equations for each age category, from toddlers to the elderly. Calculation of the physical activity level (PAL) took place by dividing the BMR predicted from the TEE by the Japanese equation [20,21]. We also calculated the BMR using the Schofield equation for further discussion [22,23].

### 2.3. Physical Activity

A triaxial accelerometer was used to monitor the daily step count (Actimarker; Panasonic, Osaka, Japan) [8]. Preschool children are forgetful, make a variety of physical movements, and they could easily break or lose their pedometers. Therefore, the accelerometers were taped onto a cardboard sheet to immobilize them and placed inside a small waist pouch (Kid’s SPIbelt**^®^**; Austin, TX, USA) [8]. This small waist pouch was made to be used by children to carry various medical monitoring devices during their daily living conditions. The pocket is secure, elastic, inconspicuous, and can carry medical monitoring supplies tightly, including an insulin pump, EpiPen, and glucose monitor. It remains in contact with their hips and can withstand bouncing movements. The bag has an adjustable strap that is compatible with the waist circumference of children and is very light. The expendable belt is applicable to a waist size of 46–68 cm. The size of the pocket is 3 × 14 cm. The pouch fastener was locked by researchers, which meant the accelerometers could not be accessed by parents and children, preventing manipulation. We ensured that the accelerometers and the hips were in close contact. We explained to the parents and preschool teachers the importance of verifying the tightness regularly throughout the study period and why we were fastening the belt of the waist bag tightly on the children’s waist. There was no significant difference between an Actimarker worn on an expendable band and one placed in the SPIbelt pouch when we compared between the methods we were using to place the accelerometer on an expandable band that is tightly placed against the hips of the young children [8]. The accelerometers were worn by the subjects between the mornings of days 0 to 9. The participants were exempted from wearing the device in special circumstances, including while wearing clothes, bathing, swimming, and when asleep, but were told to wear the device at all other periods. We analyzed the subjects’ data for seven days (days 1 to 8) consecutively, except the first day that accelerometers began being worn or when they were removed. We excluded and treated data sets beyond 30,000 steps/day and below 1000 as missing, as in previous studies. Non-valid days were also excluded based on previous recommendations [8].

The relationship between METs and accelerometer output varies between preschool children and adults [24,25,26]. By multiplying by 0.73, we corrected this difference. Categorization of Physical activity was an MVPA (≥3.0 METs) or an LPA (1.5–2.9 METs). The defined exercise duration was ≥4.0 METs of physical activity [7].

### 2.4. Statistical Analyses

Except where stated, results are submitted as mean ± standard deviation (SD). After adjusting for age, as it is an important factor during the fast maturation phase of children, analysis of covariance (ANCOVA) was used to investigate contrast in the sexes [27,28]. Partial correlation analysis was applied to examine factors related to TEE, with the adjusting variables being sex, age, height, weight, FFM, and FM or fat percentage. FFM and FM were used as covariates for standardization, for there is a significant difference from zero by the linear regression intercept of TEE towards FFM or FM. For the prediction of outcomes based on TEE, FFM, and daily step count, these were used in the equation of several linear regressions at the same time. Prompt regression analysis was carried out using age, weight, height, body mass index (BMI), %fat, FFM, and TEE as the dependent variable and the daily step count as the independent variable, to confirm the result. Pearson’s correlation coefficient was obtained to measure the correlation across the accelerometers. The α was set at *p* < 0.05. IBM SPSS Statistics for Windows, set as the thresholds for statistical significance (Version 22.0 (IBM Corp., Armonk, NY, USA) was used for all statistical investigations.

## 3. Results

### 3.1. Physical Characteristics

Table 1 presents the participants’ body composition, physical characteristics, daily step count, and energy expenditure. The mean ± SD of FFM, weight, TEE, and step count were 14.3 ± 1.9 kg, 18.0 ± 2.4 kg, 1343 ± 170 kcal/d, and 14,742 ± 4083 steps/day, respectively.

### 3.2. Sex Differences

Age was a covariate (mean age of 5.2 years) when obtaining the standard error of the mean and the marginal mean (SEM), as presented in Table 2. Girls and boys did not differ significantly regarding height, weight, BMI, FFM, TEE, %fat, PAL, or LPA. Boys had significantly higher TBW, FFM, TEE, WT, step counts, MVPA, and exercise duration than girls.

### 3.3. Determinants of TEE

**Figure 1:**
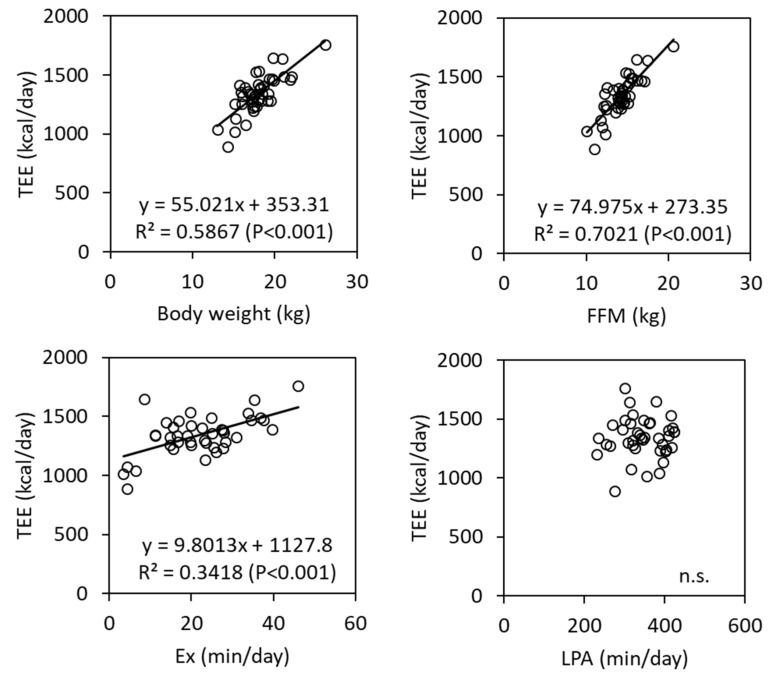
Scatter plots between total energy expenditure (TEE) and body weight, fat free mass (FFM), exercise duration (Ex), and light-intensity activity (LPA).

TEE correlation with body weight, FFM, duration of exercise, and LPA is illustrated in Figure 1. TEE significantly correlated with exercise duration, body weight, and FFM, whereas LPA did not. We used TEE as the dependent variable to conduct stepwise multiple linear regression analysis (Table 3). Sex, age, height, weight, FFM, FM, fat percentage, step count, LPA, MVPA, and exercise duration acted as the potential predictors. The significant predictors of TEE selected were step count and FFM. Table 3 presents 95% confidence intervals and the regression coefficients. The equation was as follows: TEE (kcal/day) = 69.4 × FFM (measured in kg) + 0.0114 × steps counted (steps/day) + 190, and 73.6% of the variance in the TEE was accounted for by this.

### 3.4. Determinants of WT

**Figure 2:**
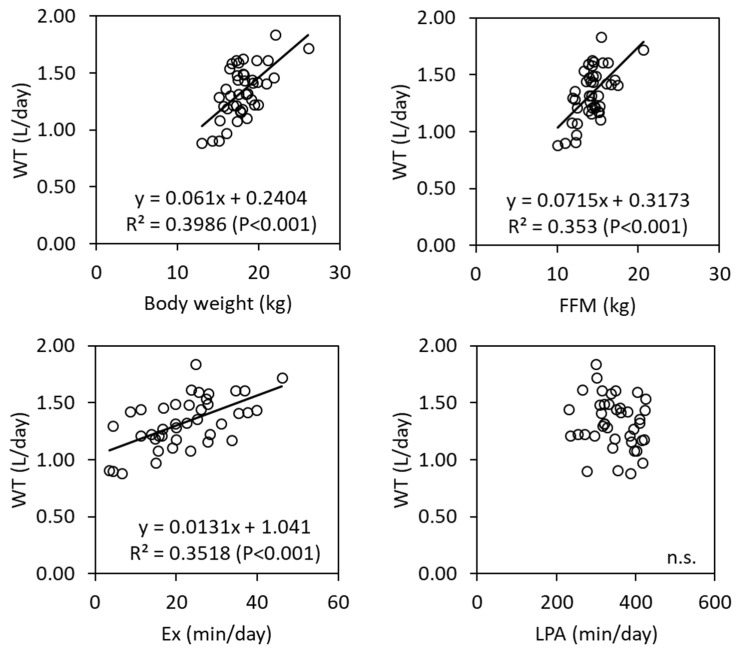
Scatter plots between water turnover (WT) and body weight, fat free mass (FFM), exercise duration (Ex), and light intensity activity (LPA).

WT and FFM, body weight, exercise duration, and LPA correlation are illustrated in Figure 2. WT significantly correlated with FFM, body weight, and exercise duration, although LPA did not. We used WT as the dependent variable to conduct stepwise multiple linear regression analysis. (Table 4). Sex, age, height, weight, FFM, FM, fat percentage, step count, LPA, and MVPA, together with exercise duration, were the potential predictors. The significant predictors of WT selected were FFM and step count. The regression coefficients and 95% confidence intervals are presented in Table 4. The equation was WT (kcal/day) = 0.043 × body weight (kg) + 0.0077 × exercise duration (min/day) + 0.385, which accounted for 47.3% of the variance in WT.

## 4. Discussion

The present study was aimed at examining WT and TEE plus their determinants in preschool children of ages 4–6 in Japan. The marginal means and SEMs of TEE and WT in boys and girls after controlling for age were 1.25 ± 0.04 and 1.42 ± 0.05 L/day, and 1287 ± 32 and 1408 ± 35 kcal/day, respectively. We confirmed that TEE is significantly determined by FFM and step count [8], and found that in Japanese preschool children, body weight and exercise duration are major determinants of WT.

Studies using DLW in young preschool children from North America and countries in Europe have been reviewed by the current Japanese Dietary Reference Intakes (Japanese DRI 2020) to tentatively establish EER, with EERs of 1300 and 1250 kcal/day for three- to five-year-old boys and girls, respectively [29]. The current EERs are similar to the present TEE values and show the usefulness of the current EER for young children in early school in Japan approximately 5 years old [8]. The present TEE values are also comparative to the most recent publication [27]. We found that TEE correlated with the FFM and step count, with the relationship being free of age, sex, height, or fat percentage. FFM and step count can be used to predict TEE. The present study showed that, in preschool children or pre-teens, FFM and step count are major influences for TEE [8,9].

WT in children has been examined by a few studies [30,31]. WT was shown to highly correlate with FFM, body weight, and TEE in children in one study [30]. However, no correlation was shown across activity energy expenditure, WT, or PAL (TEE/BMR) in children aged 5–14 in another study [30]. The indication of this study is consistent with previous studies that showed that WT highly correlates with body weight, FFM, and TEE in children. The present study also indicated that exercise duration correlates significantly with WT, whereas LPA does not. Furthermore, we found that in Japanese preschool children, body weight and exercise duration are major determinants of WT. Exercise intensity and duration determine WT in children rather than simple PAL.

Estimated TEE is authenticated against DLW and indirect calorimetry in groups of grown-ups and can be calculated by Actimarker [10,32,33]. However, the relationship between accelerometer output and METs has been demonstrated by a series of previous studies, and the ratio of accelerometer output and energy expenditure was different between adults, pre-teens, and preschool children [24,25,26]. By multiplying by 0.73, we corrected for these differences in preschool children. Care should be taken when using accelerometers to acquire data in children, as the potential influence factor is dependent on the accelerometer. The Japanese Industrial Standards for Pedometers were adhered to in the manufacture of the accelerometers used in this study (JIS S7200-1993; president of the committee, Dr. Yoshiro Hatano), and they were trialed with a maximum acceptable error of ±3% and an oscillation generator providing an acceleration of 2.4 and 4.9 m/s^2^ r. However, based on a given algorithm, resolving the true step count of young children from accelerometer output data may be difficult. There is no instrument for the assessment of physical activity that can be used for all populations, events, and research questions, according to indications from previous studies.

The major limitation of the current study is the number of participants. The generalization may be limited if this equation was not validated in a second independent sample. Overfitting the equation model can be an issue. Secondly, we did not examine any motor skills or physical fitness in the current study. The relationship between physical activity, motor skills, and physical fitness is an important issue in preschool children. Further studies are needed.

## 5. Conclusions

To guide the AI of water and EER in Japanese preschool children, we established predictive equations for WT and TEE using body weight, FFM, step count, and exercise duration: WT (L/day) = 0.043 × body weight (kg) + 0.0077 × exercise duration (min/day) + 0.385 and TEE (kcal/day) = 69.4 × FFM (measured in kg) + 0.0114 × counted steps (*n*/day) + 190. The present study found that body weight and exercise duration are major determinants of WT and asserted that step count and FFM are big influences of TEE in young preschool children in Japan.

## Figures and Tables

**Table 1:** Physical characteristics and physical activities of the participants (*n* = 41).

Characteristic	Mean	±	SD
Age (years)	5.2	±	0.9
Height (cm)	107.6	±	6.9
Weight (kg)	18.0	±	2.4
BMI (kg/m^2^)	15.5	±	1.3
TBW (kg)	10.4	±	1.4
FFM (kg)	14.3	±	1.9
%fat (%)	20.6	±	4.3
TEE (kcal/day)	1343	±	170
WT (L/day)	1.33	±	0.22
BMR_Japanese_	959	±	129
BMR_Schofield_	879	±	60
PAL	1.41	±	0.12
Step count	14,401	±	3319
LPA (1.5–2.9 METs) (min/day)	345	±	54
MVPA (≥3.0 METs) (min/day)	46	±	14
Ex (≥4.0 METs) (min/day)	22	±	10

BMI, body mass index; TBW, total body water; FFM, fat-free mass; %fat, percent body fat; TEE, total energy expenditure; BMR_Japanese_, predicted basal metabolic rate calculated using the equation for the Japanese population; BMR_Schofield_, predicted basal metabolic rate calculated using the Schofield equation; PAL, physical activity level based on BMR_Japanese_; LPA, light-intensity physical activity; MVPA, moderate-to-vigorous physical activity; Ex, Exercise duration.

**Table 2:** Comparison between girls and boys using ANCOVA adjusted for age.

Characteristic	Girls (*n* = 22)			Boys (*n* = 19)			*p* Value
	Mean	±	SEM	Mean	±	SEM	
Height (cm)	107.1	±	0.9	108.3	±	0.9	0.391
Weight (kg)	17.5	±	0.4	18.5	±	0.5	0.118
BMI (kg/m^2^)	15.2	±	0.3	15.8	±	0.3	0.164
TBW (kg)	10.0	±	0.2	10.9	±	0.2	0.008 **
FFM (kg)	13.7	±	0.3	14.9	±	0.3	0.008 **
%fat (%)	21.6	±	0.9	19.4	±	1.0	0.11
TEE (kcal/day)	1287	±	32	1408	±	35	0.019 *
WT (L/day)	1.25	±	0.04	1.42	±	0.05	0.015 *
BMR_Japanese_	910	±	24	1016	±	26	0.006 **
BMR_Schofield_	840	±	10	925	±	10	<0.001 ***
PAL	1.42	±	0.03	1.39	±	0.03	0.63
Step count	12,808	±	647	16,246	±	700	0.001 **
LPA (1.5–2.9 METs) (min/day)	348	±	12	341	±	13	0.706
MVPA (≥3.0 METs) (min/day)	41	±	3	52	±	3	0.011 *
Ex (≥4.0 METs) (min/day)	17	±	2	28	±	2	<0.001 ***

Mean and standard error of the mean (SEM) were estimated as the marginal mean with a covariate appearing in the model at the following value: age = 5.2. *p*-values in bold show significant differences between girls and boys calculated using ANCOVA; * *p* < 0.05, ** *p* < 0.01, *** *p* < 0.001.

**Table 3:** Regression analysis for predicting TEE (kcal/day).

Predictor Variable	B	β	*p*-Value	95% CI for B
FFM (kg)	69.4	0.776	<0.001	(54.1, 84.7)
Step count (*n*/day)	0.0114	0.222	0.013	(0.0026, 0.0202)
(Constant)	190		0.09	(−31, 411)

The dependent variable was TEE, total daily energy expenditure (kcal/day). B, unstandardized regression coefficient; β, standardized regression coefficient; CI, confidence interval. Stepwise regression analysis was used: sex, age, height, weight, fat mass, percentage body fat, and exercise duration were not included in the model based on the criteria of 0.05 entry and 0.10 removal for the probability of F. R^2^ = 0.749 and adjusted R^2^ = 0.736.

**Table 4:** Regression analysis for predicting WT (L/day).

Predictor Variable	B	β	*p*-Value	95% CI for B
Body weight (kg)	0.043	0.456	0.002	(0.017, 0.069)
Exercise duration (min/day)	0.0077	0.348	0.015	(0.002, 0.014)
(Constant)	0.385		0.068	(−0.03, 0.8)

The dependent variable was WT, water turnover (L/day). B, unstandardized regression coefficient; β, standardized regression coefficient; CI, confidence interval. Stepwise regression analysis was used: sex, age, height, fat-free mass, fat mass, percentage body fat, and step count were not included in the model based on the criteria of 0.05 entry and 0.10 removal for the probability of F. R^2^ = 0.500 and adjusted R^2^ = 0.473.

## Data Availability

The data presented in this study are available on request from the corresponding author. The data are not publicly available due to ethical reason.

## References

[1] Pontzer H., Brown M.H., Wood B.M., Raichlen D.A., Mabulla A.Z.P., Harris J.A., Dunsworth H., Hare B., Walker K., Luke A. (2021). Evolution of water conservation in humans. Curr. Biol. CB.

[2] Westerterp K.R., Plasqui G., Goris A.H. (2005). Water loss as a function of energy intake, physical activity and season. Br. J. Nutr..

[3] Rosinger A.Y. (2020). Biobehavioral variation in human water needs: How adaptations, early life environments, and the life course affect body water homeostasis. Am. J. Hum. Biol. Off. J. Hum. Biol. Counc..

[4] WHO (2017). The Double Burden of Malnutrition. Policy Brief.

[5] Raman A., Schoeller D.A., Subar A.F., Troiano R.P., Schatzkin A., Harris T., Bauer D., Bingham S.A., Everhart J.E., Newman A.B. (2004). Water turnover in 458 American adults 40–79 year of age. Am. J. Physiol. Ren. Physiol..

[6] Speakman J.R., Yamada Y., Ainslie P.N. (2021). Adopting a standard calculation for human doubly labeled water studies. Cell Rep Med..

[7] Miyachi M. (2012). Measures of physical activity and exercise for health promotion by the Ministry of Health, Labour and Welfare. J. Phys. Fit. Sports Med..

[8] Yamada Y., Sagayama H., Itoi A., Nishimura M., Fujisawa K., Higaki Y., Kimura M., Aoki Y. (2020). Total Energy Expenditure, Body Composition, Physical Activity, and Step Count in Japanese Preschool Children: A Study Based on Doubly Labeled Water. Nutrients.

[9] Komura K., Nakae S., Hirakawa K., Ebine N., Suzuki K., Ozawa H., Yamada Y., Kimura M., Ishii K. (2017). Total energy expenditure of 10- to 12-year-old Japanese children measured using the doubly labeled water method. Nutr. Metab..

[10] Yamada Y., Hashii-Arishima Y., Yokoyama K., Itoi A., Adachi T., Kimura M. (2018). Validity of a triaxial accelerometer and simplified physical activity record in older adults aged 64-96 years: A doubly labeled water study. Eur. J. Appl. Physiol..

[11] Yamada Y., Yokoyama K., Noriyasu R., Osaki T., Adachi T., Itoi A., Naito Y., Morimoto T., Kimura M., Oda S. (2009). Light-intensity activities are important for estimating physical activity energy expenditure using uniaxial and triaxial accelerometers. Eur. J. Appl. Physiol..

[12] Racette S.B., Schoeller D.A., Luke A.H., Shay K., Hnilicka J., Kushner R.F. (1994). Relative dilution spaces of 2H- and 18O-labeled water in humans. Am. J. Physiol. Endocrinol. Metab..

[13] Sagayama H., Yamada Y., Racine N.M., Shriver T.C., Schoeller D.A. (2016). Dilution space ratio of 2H and 18O of doubly labeled water method in humans. J. Appl. Physiol..

[14] Schoeller D.A., Ravussin E., Schutz Y., Acheson K.J., Baertschi P., Jequier E. (1986). Energy expenditure by doubly labeled water: Validation in humans and proposed calculation. Am. J. Physiol. Regul. Integr. Comp. Physiol..

[15] Black A.E., Prentice A.M., Coward W.A. (1986). Use of food quotients to predict respiratory quotients for the doubly-labelled water method of measuring energy expenditure. Hum. Nutr. Clin. Nutr..

[16] Weir J.B. (1949). New methods for calculating metabolic rate with special reference to protein metabolism. J. Physiol..

[17] International Atomic Energy Agency (2009). IAEA Human Health Series No. 3. Assessment of Body Composition and Total Energy Expenditure in Humans Using Stable Isotope Techniques.

[18] International Atomic Energy Agency (2011). Iaea Human Health Series no. 13 Introduction to Body Composition Assessment Using the Deuterium Dilution Technique with Analysis of Urine Samples by Isotope Ratio Mass Spectrometry.

[19] Health Promotion and Nutrition Division-Health Service Bureau Ministry of Health and Welfare (1995). Recommended Dietary Allowances for the Japanese.

[20] Westerterp K.R. (2013). Physical activity and physical activity induced energy expenditure in humans: Measurement, determinants, and effects. Front. Physiol..

[21] Westerterp K.R. (2018). Exercise, energy expenditure and energy balance, as measured with doubly labelled water. Proc. Nutr. Soc..

[22] Sentongo T.A., Tershakovec A.M., Mascarenhas M.R., Watson M.H., Stallings V.A. (2000). Resting energy expenditure and prediction equations in young children with failure to thrive. J. Pediatrics.

[23] Schofield W.N. (1985). Predicting basal metabolic rate, new standards and review of previous work. Hum. Nutr. Clin. Nutr..

[24] Ohkawara K., Oshima Y., Hikihara Y., Ishikawa-Takata K., Tabata I., Tanaka S. (2011). Real-time estimation of daily physical activity intensity by a triaxial accelerometer and a gravity-removal classification algorithm. Br. J. Nutr..

[25] Tanaka C., Hikihara Y., Ando T., Oshima Y., Usui C., Ohgi Y., Kaneda K., Tanaka S. (2019). Prediction of Physical Activity Intensity with Accelerometry in Young Children. Int. J. Environ. Res. Public Health.

[26] Hikihara Y., Tanaka C., Oshima Y., Ohkawara K., Ishikawa-Takata K., Tanaka S. (2014). Prediction models discriminating between nonlocomotive and locomotive activities in children using a triaxial accelerometer with a gravity-removal physical activity classification algorithm. PLoS ONE.

[27] Pontzer H., Yamada Y., Sagayama H., Ainslie P.N., Andersen L.F., Anderson L.J., Arab L., Baddou I., Bedu-Addo K., Blaak E.E. (2021). Daily energy expenditure through the human life course. Science.

[28] Westerterp K.R., Yamada Y., Sagayama H., Ainslie P.N., Andersen L.F., Anderson L.J., Arab L., Baddou I., Bedu-Addo K., Blaak E.E. (2021). Physical activity and fat-free mass during growth and in later life. Am. J. Clin. Nutr..

[29] Ministry of Health Labour and Welfare Japan (2020). Japanese Dietary Reference Intake.

[30] Rush E.C., Chhichhia P., Kilding A.E., Plank L.D. (2010). Water turnover in children and young adults. Eur. J. Appl. Physiol..

[31] Fusch C., Hungerland E., Scharrer B., Moeller H. (1993). Water turnover of healthy children measured by deuterated water elimination. Eur. J. Pediatrics.

[32] Murakami H., Kawakami R., Nakae S., Yamada Y., Nakata Y., Ohkawara K., Sasai H., Ishikawa-Takata K., Tanaka S., Miyachi M. (2019). Accuracy of 12 Wearable Devices for Estimating Physical Activity Energy Expenditure Using a Metabolic Chamber and the Doubly Labeled Water Method: Validation Study. JMIR mHealth uHealth.

[33] Matsumura Y., Yamamoto M., Kitado T., Nakamura H., Kidera K., Fujimoto S. (2008). High-accuracy physical activity monitor utilizing three-axis accelerometer. Natl. Tech. Rep..

